# Scalable Multi‐Target Illusion via a Segmented Spatiotemporal Metasurface

**DOI:** 10.1002/nap2.70316

**Published:** 2026-10-11

**Authors:** Yujie Hong, Dan Liu, Yi Qian, Guangzheng Ren, Wenlong Li, Hailin Huang, Shangjie Chen, Cheng Huang, Huilin Mu, Chunsheng Guan, Tong Cai

**Affiliations:** ^1^ Air and Missile Defense College Air Force Engineering University Xi'an China; ^2^ Fundamentals Department Air Force Engineering University Xi'an China

**Keywords:** multi‐target illusion generation, parallel‐sequence spectral translation, perception reconstruction, spatial segmentation

## Abstract

A kaleidoscope converts a few physical fragments into numerous images through repeated mirror reflections, yielding visual scene richer than the objects themselves. This optical phenomenon shows that a perceived scene can be reconstructed by beam redirection and spatial reorganization. Inspired by this principle, we propose a *scalable multi‐target illusion* based on a *segmented spatiotemporal metasurface*. It acts as an electromagnetic kaleidoscope and operates through the proposed *parallel‐sequence spectral translation mechanism* (PSTM): one physical aperture is partitioned into independently driven segments, each loaded with a distinct time‐varying bias sequence. Under linear frequency‐modulated signal illumination, PSTM translates the echo spectrum into multiple independently coded frequency‐shifted components. After signal processing by the detector, these sequence‐translated spectral components are reconstructed as scalable false targets in the range–velocity plane. Moreover, the reflection amplitudes of individual targets can be further tuned by aperture allocation. A programmable metasurface is designed and fabricated to validate the concept. Experiments demonstrate the generation of two, three, four, and five false targets, with the measured range–velocity features closely matching theoretical predictions. This work extends metasurface‐based illusion from single‐response modulation to *scalable multi‐target scene reconstruction* with controllable multidimensional features, providing a flexible framework for electromagnetic illusion generation from a single physical aperture.

## Graphical abstract

An electromagnetic kaleidoscope is realized with a segmented spatiotemporal metasurface that transforms one physical aperture into multiple independently controlled false targets. Parallel‐sequence spectral translation codes apparent range and velocity, while segment allocation regulates echo amplitude. Experiments at 5 GHz generate two to five targets with range–velocity features closely matching theoretical predictions.

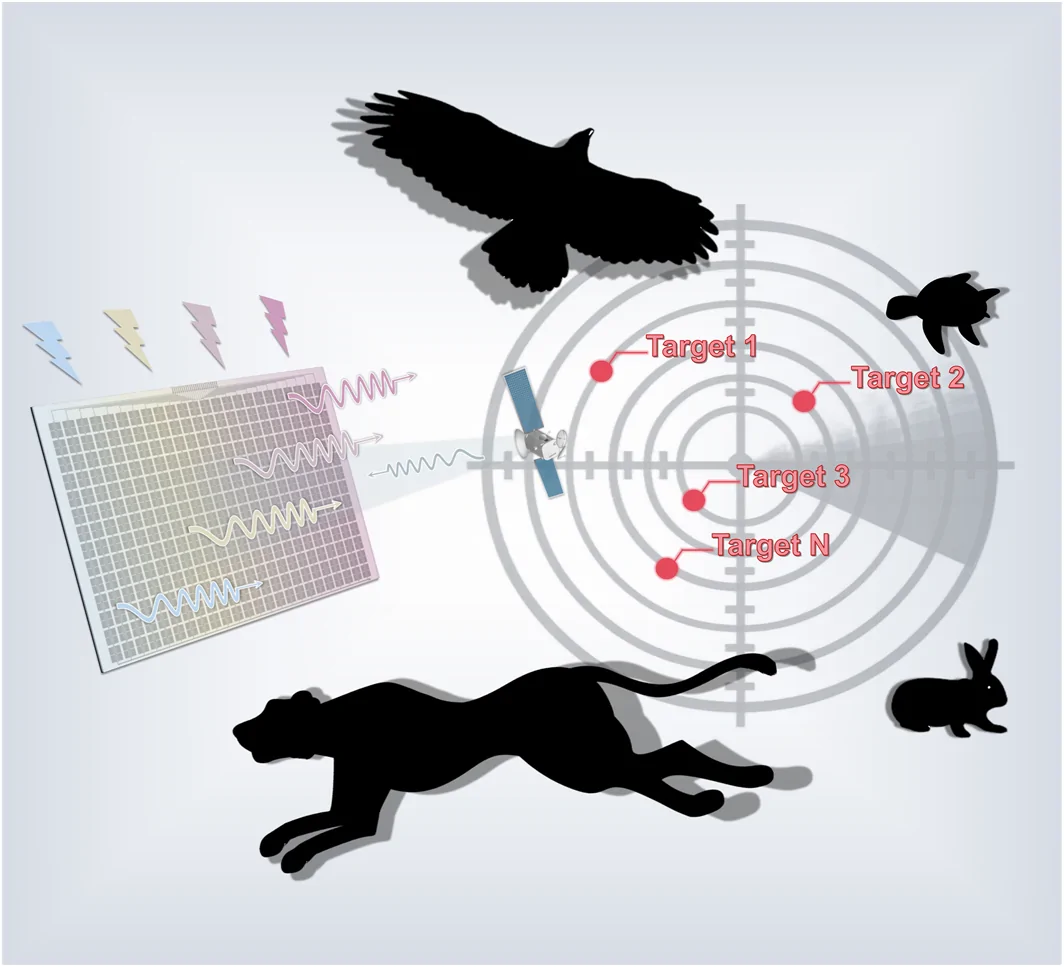

## 1 Introduction

In visual perception, the number and distribution of observed objects can be substantially shaped by the surrounding optical environment. A typical example is the kaleidoscope, where mirror reflections reorganize a small number of physical fragments into abundant apparent images with ordered spatial distributions. The observer therefore perceives a scene that is not a direct copy of the objects inside, but a reconstructed result produced by beam redirection and spatial multiplication. This phenomenon provides an intuitive analogy for electromagnetic perception. Detection systems also infer a target scene from the returned signal rather than directly observing the physical object itself. Under a linear frequency‐modulated (LFM) signal, the range and velocity of a target are reconstructed from the beat‐frequency distribution and coherent phase evolution of the received echoes [1, 2]. Therefore, once the echo channels can be redirected and encoded in a programmable manner, the perceived scene can be reshaped beyond the actual physical target, enabling artificial target‐like responses with prescribed range–velocity features [3, 4, 5].

Conventional deception techniques mainly rely on active electronic systems, such as delay modulation, frequency translation, digital radio‐frequency memory, and coherent retransmission. These approaches can flexibly synthesize false target information, but they often require complicated radio‐frequency chains, high‐speed signal acquisition and reconstruction, precise synchronization, and considerable power consumption. Metasurfaces offer an alternative route for manipulating electromagnetic echoes with a much thinner and more compact architecture. By arranging subwavelength meta‐atoms with tailored electromagnetic responses, metasurfaces can control the phase, amplitude, polarization, and wavefront of scattered waves with high spatial resolution [6, 7, 8, 9, 10, 11, 12, 13, 14]. The development of programmable, digital‐coding, and time‐modulated metasurfaces has further expanded this capability from static wavefront shaping to dynamic spectral manipulation during wave–matter interaction [15, 16, 17, 18, 19, 20, 21, 22, 23, 24, 25, 26, 27, 28, 29, 30, 31]. As a result, metasurface‐based deception has achieved false‐range generation, false‐velocity generation, SAR imaging jamming, moving‐target jamming, and joint range–Doppler modulation [32, 33, 34, 35, 36, 37, 38, 39, 40, 41, 42, 43, 44, 45, 46]. Although these studies have greatly enriched the capability of metasurface‐enabled deception, most reported schemes still operate within a single‐response framework [32, 33, 34, 35, 36, 37, 38, 39, 42, 44, 45]. In such systems, the whole metasurface aperture usually behaves as one equivalent modulation channel, and the generated deception effect is dominated by one false target, one spectral shift, or one principal scattering response. This operation mode is suitable for single‐target illusion, but it cannot readily construct a complex scene containing multiple independently controllable false targets. In contrast, a kaleidoscopic perception requires spatial multiplication and differentiated encoding among different image channels. Translating this idea into radar deception requires a metasurface architecture in which one physical aperture can be divided into multiple parallel echo‐control channels, with each channel carrying an independent modulation code and contributing to an independently positioned target response in the range–velocity plane.

In this paper, we propose a *scalable multi‐target illusion* strategy based on a *segmented spatiotemporal* metasurface. The core of the proposed strategy is a *parallel‐sequence spectral translation mechanism* (PSTM), which converts one physical scattering aperture into multiple parallel sequence‐coded spectral channels through segment‐level temporal modulation. As illustrated in Figure 1, the proposed scheme uses segment arrangement, rather than independent physical reflectors, to construct multiple controllable echo channels within a single aperture. Each segment carries different modulation‐induced spectral characteristics. In the implementation, the desired multiple false‐target coordinates (*R*_*q*_, *v*_*q*_) are first specified, and the corresponding fast‐time and slow‐time modulation frequencies are then determined according to the range–velocity mapping relation. These modulation frequencies are converted into phase sequences and further translated into practical bias sequences through the measured voltage–phase response of the tunable meta‐atoms. A 24 × 14 programmable metasurface with tunable reflection responses over 4.3–5.5 GHz is designed and fabricated, and 5 GHz is selected to verify this mechanism. By loading different bias sequences onto different segments, controllable two‐, three‐, four‐, and five‐target illusions are experimentally generated in the range–velocity plane, demonstrating that PSTM enables one physical aperture to be perceived as multiple designed false targets with controllable range, velocity, and amplitude features.

**FIGURE 1:**
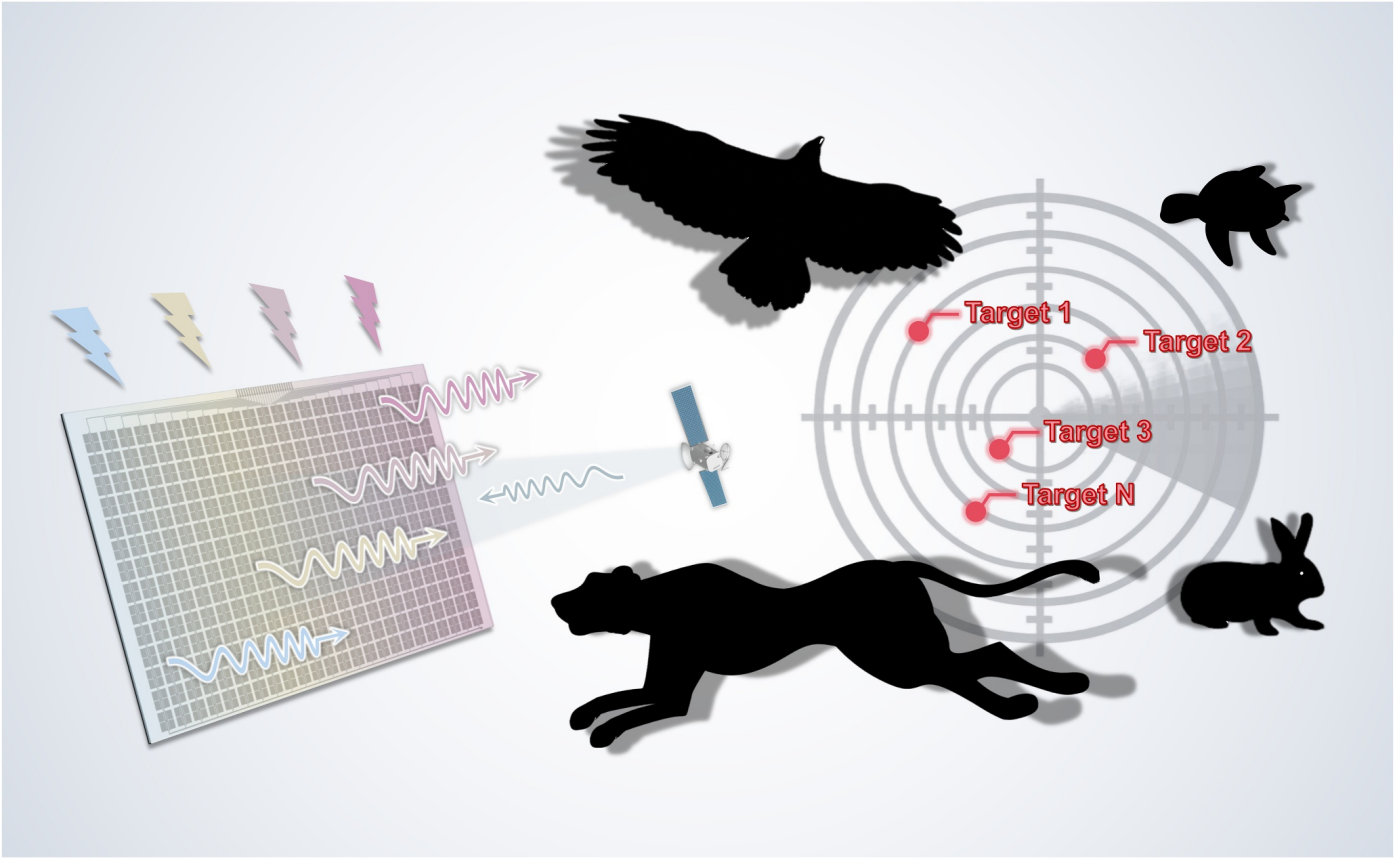
Concept of scalable multi‐target illusion based on a segmented spatiotemporal metasurface. A programmable metasurface is divided into multiple independently modulated segments, and each segment is driven by a distinct time‐varying bias sequence and produces a reflected echo component with a different frequency shift *f*_*m*,1_, *f*_*m*,2_, *f*_*m*,3_, …, *f*_*m*,*N*_ when illuminated by a detection signal. These frequency‐shifted components are coherently received and processed by the detector, producing multiple false target responses with different apparent range and velocity parameters (*R*_1_, *v*_1_), (*R*_2_, *v*_2_), …, (*R*_*N*_, *v*_*N*_) in the perception plane with a single metasurface.

## 2 Working Principle for Multi‐Target Illusion Generation

The principle of the proposed multi‐target illusion generation strategy is illustrated in Figure 2. The designed metasurface aperture is divided into multiple independently driven segments, and each segment is assigned a distinct time‐varying bias sequence. In this way, the whole metasurface no longer behaves as a single equivalent reflection channel. Instead, it forms a set of parallel sequence‐coded modulation channels, each of which produces a reflected echo component with a prescribed spectral translation under the same LFM illumination. This process is described here as the parallel‐sequence spectral translation mechanism. In PSTM, the scattering response of one physical metasurface is decomposed into multiple segment‐dependent translated spectral components through segment‐level temporal modulation. The role of PSTM is to translate the spectrum of the reflected echoes before radar processing, while the subsequent dechirp and two‐dimensional Fourier processing resolve these translated spectral components as multiple target‐like responses. Figure 2a shows the segment arrangement and independent modulation of the metasurface. Different segments are loaded with different bias sequences, so that the reflected echoes carry different modulation‐induced spectral features. Figure 2b illustrates the spectral‐translation result of the modulated echoes. Owing to the time‐varying reflection phase imposed on each segment, the echo spectrum generated by the *q*th segment is translated to a prescribed frequency‐shifted component. Multiple segments therefore produce multiple distinguishable translated spectral components under the same incident LFM signal. After dechirp processing and two‐dimensional fast Fourier transformation by the detector, these translated spectral components are resolved as distinct peaks in the range–velocity plane, corresponding to multiple false targets, as shown in Figure 2c. Different from a single‐tone frequency‐shift modulation applied to the whole aperture, PSTM assigns segment‐dependent spectral codes to parallel sequence‐coded modulation channels. The frequency code assigned to the *q*th segment is described by a composite modulation code *f*_*m*,*q*_, which contains two functional components: the fast‐time component *f*_*R*,*q*_, which modifies the beat frequency after dechirp processing and therefore determines the apparent range, and the slow‐time component *f*_*D*,*q*_, which controls the pulse‐to‐pulse phase progression and therefore determines the apparent velocity. Meanwhile, the aperture area allocated to each segment introduces an additional amplitude‐control degree of freedom, because a larger segment contains more coherently modulated meta‐atoms and produces a stronger frequency‐shifted echo component. The PSTM relation can be written from the modulation side as

(1) $$\Gamma_{\text{PSTM}}\left(x,y,t,n\right)=\sum_{q=1}^{N}\chi_{q}\left(x,y\right)\alpha_{q}\;\exp\left\{j2\pi \left[f_{R,q}\left(t-nT_{p}\right)+f_{D,q}t\right]\right\},$$

where *χ*_*q*_ (*x*, *y*) selects the *q*th segment and *α*_*q*_ represents its effective amplitude weight. Other parameters and the detailed derivation are provided in Supporting Information S1: Note 1. Equation (1) shows that PSTM transforms one physical metasurface response into multiple segment‐resolved translated spectral components with independently assigned frequency shifts and amplitude weights.

**FIGURE 2:**
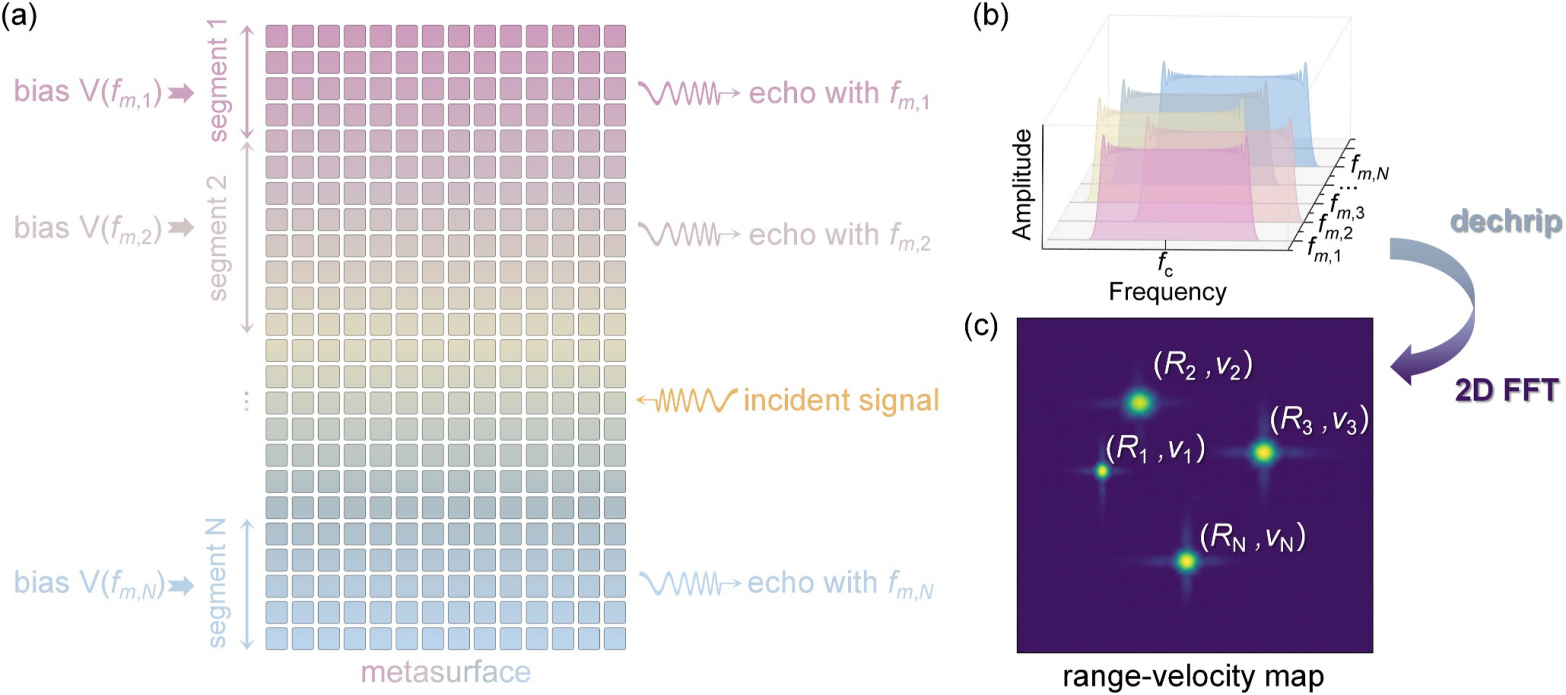
Principle of scalable multi‐target illusion via a segmented spatiotemporal metasurface. (a) Segment arrangement and independent modulation of the metasurface. The metasurface aperture is divided into *N* independently driven segments, and the *q*th segment is loaded with a bias sequence *V* (*f*_*m*,*q*_), producing an echo component with a prescribed frequency shift *f*_*m*,*q*_ under the same incident detection signal. (b) Spectral translation of the modulated echoes. The echoes generated by different segments are translated to multiple distinguishable spectral components according to their assigned modulation frequencies. (c) Reconstructed range–velocity map after dechirp processing and two‐dimensional fast Fourier transformation. The translated spectral components are resolved into different positions in the range–velocity plane, corresponding to multiple designed false targets (*R*_1_, *v*_1_), (*R*_2_, *v*_2_), …, (*R*_*N*_, *v*_*N*_). This process links segment arrangement, parallel sequence coding, spectral translation, and range–velocity processing into a complete multi‐target illusion generation chain.

By deriving the mapping between the modulation frequencies and the apparent target coordinates (see Supporting Information S1: Note 1), the false target generated by the *q*th segment appears at

(2) $$R_{q}=R_{0}+\frac{cf_{R,q}}{2K},$$

(3) $$v_{q}=v_{0}+\frac{\lambda f_{D,q}}{2},$$

where *R*_0_ and *v*_0_ are the physical range and physical radial velocity of the metasurface, respectively, *K* is the chirp slope of the LFM signal, *c* is the speed of light in free space, and *λ* is the operating wavelength. These two relations indicate that *f*_*R*,*q*_ and *f*_*D*,*q*_ provide two independent spectral‐control handles for positioning the false target in the range and velocity dimensions.

After dechirp processing and two‐dimensional fast Fourier transformation, the reconstructed response can be approximated as

(4) $$S_{\text{PSTM}}\left(R,v\right)\approx \sum_{q=1}^{N}\alpha_{q}P_{R}\left(R-R_{q}\right)P_{V}\left(v-v_{q}\right),$$

where *P*_*R*_ (·) and *P*_*V*_ (·) are the point‐spread functions in the range and velocity dimensions, respectively. Equation (4) gives the detector‐reconstructed result of the translated spectral components produced by PSTM. The peak location is determined by the spectral code (*f*_*R*,*q*_, *f*_*D*,*q*_), while the peak intensity is weighted by *α*_*q*_, which is mainly governed by the segment allocation.

Therefore, the proposed PSTM establishes a direct connection between segment‐level sequence design and multi‐target illusion generation. Segment arrangement determines the number and layout of available modulation channels, temporal spectral coding determines the reconstructed target coordinates, and segment allocation regulates the relative target amplitudes. By assigning different *f*_*R*,*q*_, *f*_*D*,*q*_, and αq to different segments, one physical metasurface can be reconstructed by the detector as multiple false targets with controllable range, velocity, and amplitude features.

## 3 Design and Characterization of the Metasurface

To implement the segmented spatiotemporal modulation described in Section 2, a tunable reflective programmable metasurface is designed and fabricated as shown in Figure 3. The metasurface exhibits tunable reflection responses from 4.3 to 5.5 GHz, and 5 GHz is selected as the validation frequency. The aperture consists of 24 × 14 tunable meta‐atoms. Figure 3a shows a photograph of the metasurface. The meta‐atoms are arranged periodically with a period of 15 mm. To enable segmented control, the aperture is divided into several independently biased modulation segments. Meta‐atoms within the same segment share one bias channel or one group of bias channels, whereas different segments are driven independently, enabling spatially differentiated temporal modulation across the aperture. This architecture allows different time‐varying phase sequences to be loaded onto different segments of the same physical aperture, thereby realizing segmented spatiotemporal modulation. Figure 3b,c show the top and side views of the tunable meta‐atom, respectively. The meta‐atom consists of a top metallic pattern, a dielectric substrate, a bottom ground plane, and bias connections. The top metallic pattern forms the reflective resonance within the frequency band of interest, whereas the varactor diode is integrated at the gap of the metallic pattern to regulate the effective electromagnetic response. The dielectric substrate is F4B, with a thickness of 2 mm, relative permittivity of 2.65, and loss tangent of 0.001.

**FIGURE 3:**
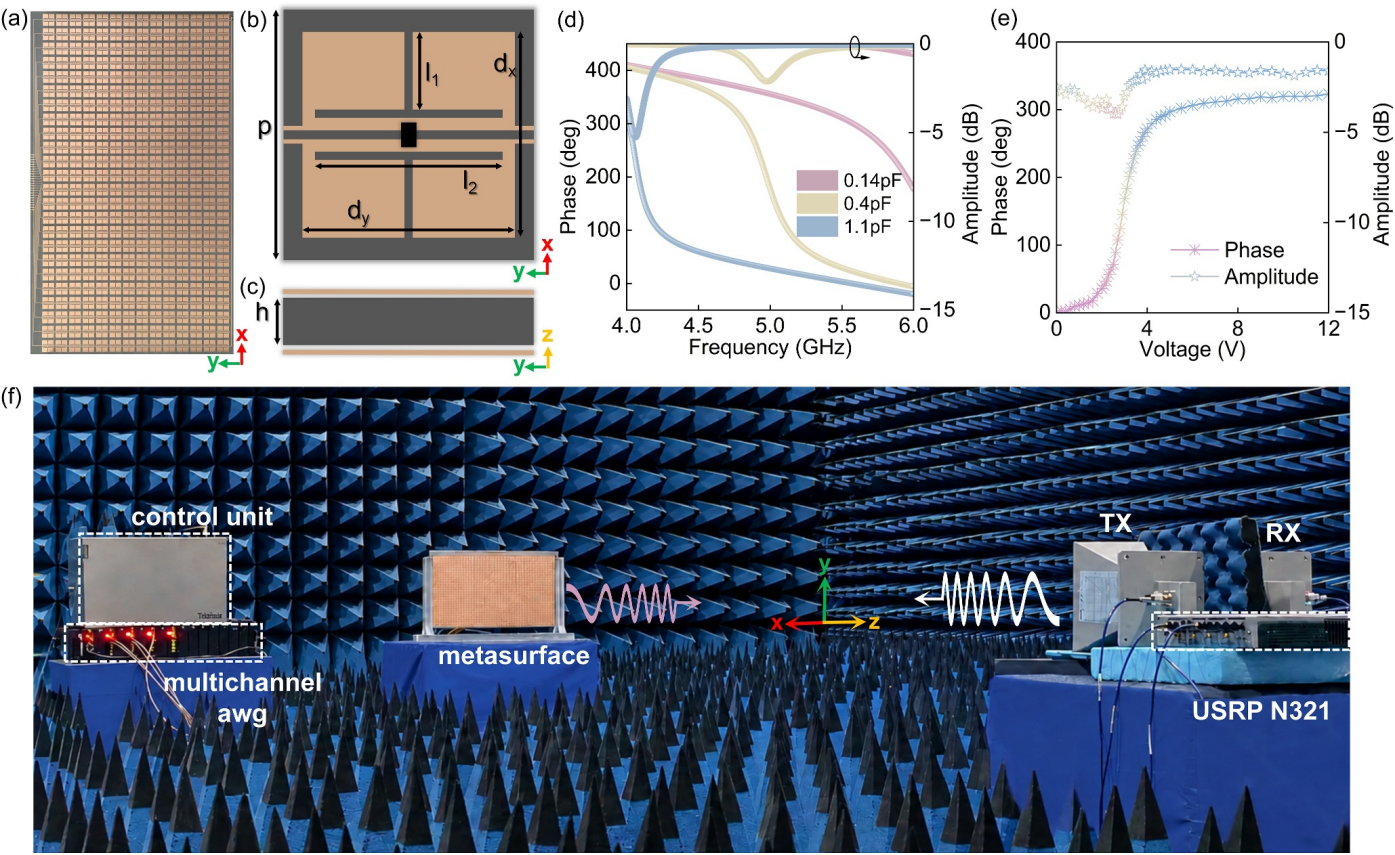
Structural parameters and electromagnetic characteristics of the meta‐atom, and experimental configuration. (a) Photograph of the fabricated metasurface. (b) Top view of the tunable meta‐atom, with geometrical parameters *p* = 15 mm, *l*_1_ = 4.5 mm, *l*_2_ = 11 mm, *d*_*x*_ = 12.5 mm, and *d*_*y*_ = 13 mm. (c) Side view of the meta‐atom (*h* = 2 mm). (d) Simulated S_11_ parameters of the meta‐atom under representative equivalent capacitance values, showing the frequency‐dependent reflection response across the operating band. (e) Measured reflection phase and amplitude as a function of applied bias voltage at 5 GHz. (f) Experimental configuration in the microwave anechoic chamber.

The MA46H120 diode is selected as the tunable component, whose equivalent capacitance varies with the applied bias voltage. Changing the bias voltage modifies the effective resonance of the meta‐atom and thereby continuously tunes the reflection phase. Figure 3d presents the S_11_ parameters of the meta‐atom. As the capacitance changes from 0.14 to 1.1 pF, the meta‐atom provides a reflection phase coverage of approximately 335°, while maintaining a relatively stable reflection amplitude. This wide and continuous phase‐tuning range provides the necessary modulation depth for synthesizing programmable time‐varying reflection responses at the operating frequency. To convert the designed phase sequences into biasing sequences, the voltage–phase response of the meta‐atom is further characterized experimentally. Figure 3e shows the measured reflection phase and amplitude as a function of the applied bias voltage at the validation frequency. As the bias voltage varies from 0 to 12 V, the reflection phase changes continuously with a coverage of approximately 335°, and the amplitude remains above −5 dB. This measured voltage–phase mapping provides the calibration basis for sequence generation. According to the PSTM relation in Section 2, the modulation frequencies assigned to the *q*th segment determine the required time‐varying phase sequence *ϕ*_*q*_ (*t*,*n*). Using the measured voltage–phase response in Figure 3e, this phase sequence is converted into the executable bias sequence as

(5) $$U_{q}\left(t,n\right)=F^{-1}\left[\phi_{q}\left(t,n\right)\right],$$

where *F* (·) denotes the measured voltage–phase mapping of the meta‐atom. In this way, the spectral codes designed in the PSTM framework are translated into practical biasing sequences that can be delivered by the control circuit. The phase coverage, monotonicity, and channel independence of the meta‐atom's voltage–phase response collectively ensure adequate modulation depth, sequence fidelity, and segment isolation, providing the hardware foundation for scalable multi‐target illusion generation. Figure 3f further presents the experimental configuration, while the detailed incident signal parameters and equipment models are described in Section 4 in direct connection with the experimental false‐target results.

## 4 Experimental Validation and Scalability Characterization of Multi‐Target Illusion Generation

The experimental configuration for multi‐target illusion generation is shown in Figure 3f. The metasurface was placed in a microwave anechoic chamber and illuminated by an LFM detection signal generated by a USRP N321 software‐defined radio platform. The transmitting and receiving antennas were connected to the TX and RX ports of the USRP N321, and both faced the metasurface, so that the USRP generated the incident LFM signal and simultaneously sampled the reflected echo. In the measurement, the initial physical distance between the metasurface and the antenna pair was set to 4 m. A multichannel arbitrary waveform generator, together with the control unit, delivered calibrated bias sequences to different segments, enabling multiple segments of the same aperture to be independently driven during acquisition. The detection signal was centered at 5 GHz, with a sweep bandwidth of 100 MHz, a chirp duration of 80 μs, a sampling rate of 250 MSa/s, and a pulse‐repetition frequency of 10 kHz. Each range–velocity frame was formed from 256 coherent chirps, and a 512‐point Doppler FFT was used for velocity–domain interpolation. Under these settings, the range resolution and range sampling interval were 1.5 and 0.6 m, respectively, while the coherent‐processing‐limited velocity resolution and the Doppler‐grid spacing were 1.17 m/s and 0.586 m/s, respectively.

### 4.1 Experimental Validation of Range–Velocity Feature Reconstruction

Figure 4 presents the experimental results of multi‐target illusion based on the PSTM. Each case comprises five panels showing the segment arrangement scheme, time‐varying phase sequences, corresponding biasing sequences, three‐dimensional range–velocity spectrum, and two‐dimensional range–velocity response, respectively. Specifically, Figure 4a–t correspond to the two‐, three‐, four‐, and five‐target experiments. The three‐dimensional spectra provide a direct view of the peak separation and amplitude distribution in the range–velocity plane, whereas the two‐dimensional maps are used to extract the target coordinates quantitatively. The experimentally measured reflection amplitude obtained under uniform full‐aperture modulation is defined as 1 and used as a common normalization reference for all cases in Figure 4. All target amplitudes are normalized using this same reference in each individual experiment. This treatment enables a direct comparison of the influence of segment division on the reflected amplitude. Across all cases, the frequency‐shifted echoes from different segments appear as distinct peaks in the range–velocity plane and be resolved as independently generated false targets. To present the experimental results more clearly, the theoretical and measured range–velocity coordinates of the generated false‐target peaks are summarized together with the modulation frequency parameters in Supporting Information S1: Note 2 and Table S1. In the two‐target experiment, the metasurface aperture was divided into two independently modulated segments, which were loaded with time‐varying modulation sequences of 0.5010 and 1.0010 MHz, respectively. As shown in Figure 4d,e, and Supporting Information S1: Table S1, the two measured false targets appear at (*R*_1_, *v*_1_) = (63.0 m, 30.09 m/s), and (*R*_2_, *v*_2_) = (123.0 m, 30.09 m/s), which agree well with the theoretical values of (64.12 m, 30.00 m/s) and (124.12 m, 30.00 m/s), respectively. This result indicates that, when the velocity–domain modulation is kept identical, different range‐modulation frequencies separate the target peaks along the range dimension. The three‐target experiment further demonstrates velocity–domain controllability. The three segments were loaded with modulation sequences of 1.5005, 1.5010, and 1.5015 MHz, respectively. As shown in Figure 4i,j, the three target peaks were reconstructed near the same range, with measured coordinates of (*R*_1_, *v*_1_) = (183.0 m, 14.92 m/s), (*R*_2_, *v*_2_) = (183.0 m, 30.09 m/s), and (*R*_3_, *v*_3_) = (183.0 m, 44.77 m/s). These results agree well with the theoretical coordinates of (184.06 m, 15.00 m/s), (184.12 m, 30.00 m/s), and (184.18 m, 45.00 m/s), verifying that the slow‐time modulation component can separate false targets along the velocity dimension when the range–domain encoding is nearly identical. In the four‐target experiment, four independently driven segments were assigned modulation sequences of 0.5008, 1.0016, 1.5024, and 2.0032 MHz. As shown in Figure 4n,o, the measured target coordinates are (*R*_1_,*v*_1_) = (63.0 m, 23.22 m/s), (*R*_2_, *v*_2_) = (123.0 m, 48.19 m/s), (*R*_3_, *v*_3_) = (184.2 m, 72.16 m/s), and (*R*_4_, *v*_4_) = (244.2 m, 96.14 m/s), respectively, matching the designed range–velocity distribution. In the five‐target experiment, five segments were driven by sequences of 0.5012, 1.0030, 1.5015, 2.0006, and 2.5032 MHz. As shown in Figure 4s,t, the corresponding measured false‐target coordinates are (*R*_1_, *v*_1_) = (63.0 m, 35.96 m/s), (*R*_2_, *v*_2_) = (124.2 m, 89.77 m/s), (*R*_3_, *v*_3_) = (183.0 m, 44.77 m/s), (*R*_4_, *v*_4_) = (243.0 m, 17.86 m/s), and (*R*_5_, *v*_5_) = (304.2 m, 96.14 m/s). To further quantify the agreement between the theoretical design and the experimental reconstruction, the relative errors between the theoretical and measured coordinates in Supporting Information S1: Table S1 were calculated for all generated false targets. The relative range error varies from approximately 0.03%–1.75%, with an average value of 0.72%, while the relative velocity error varies from approximately 0.05%–3.25%, with an average value of 0.67%. These small relative errors confirm that the reconstructed false‐target features agree well with the theoretical predictions. The largest relative velocity error occurs at the velocity of 24.00 m/s; nevertheless, its absolute deviation is only 0.78 m/s, which remains smaller than the coherent‐processing‐limited velocity resolution of 1.17 m/s. The residual deviations are mainly associated with the finite range and Doppler sampling grids used for peak extraction. Additional practical factors, including voltage–phase calibration accuracy, modulation‐sequence discretization, measurement noise, and possible inter‐segment coupling, may also contribute to the small discrepancies between the theoretical and measured coordinates.

**FIGURE 4:**
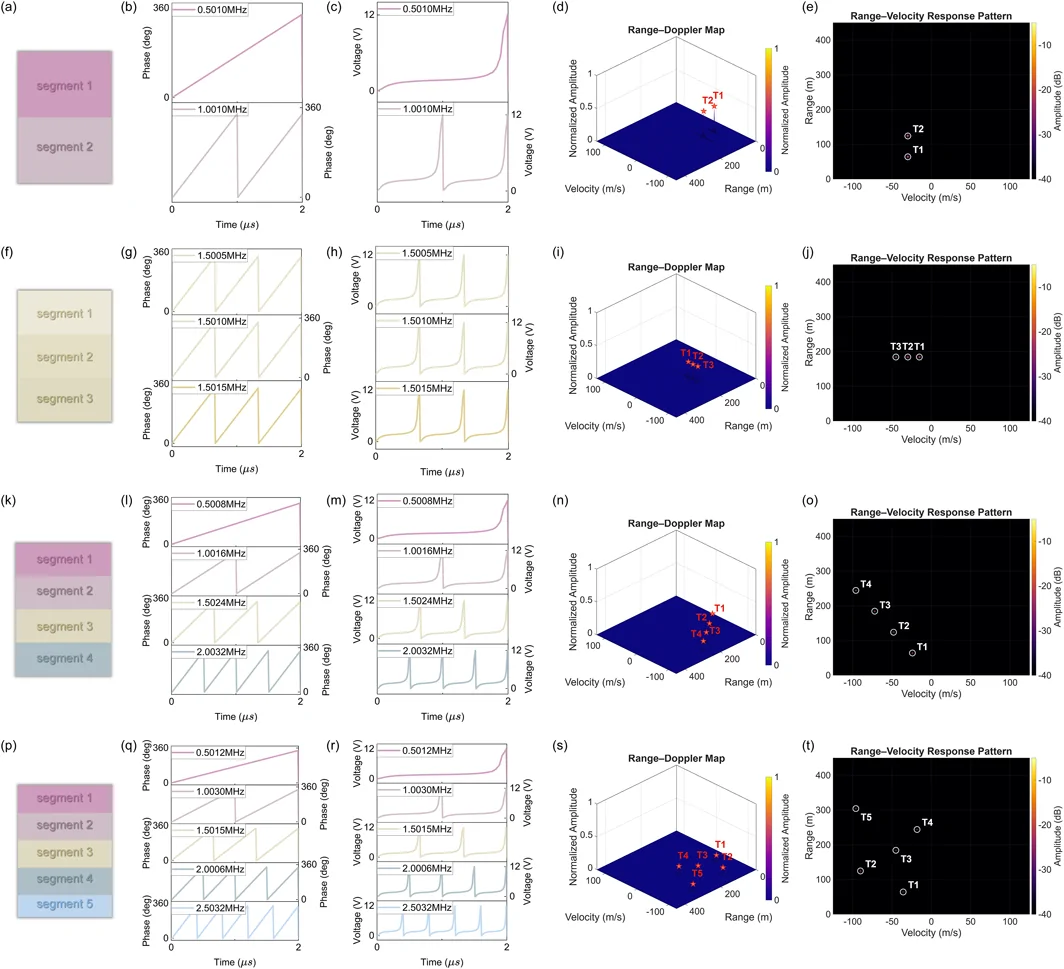
Experimental demonstration of scalable multi‐target illusion generation. Each case comprises the segment arrangement scheme, phase sequence, bias sequence, three‐dimensional range–velocity map, and two‐dimensional range–velocity response. (a–e) Two‐target case. (f–j) Three‐target case. (k–o) Four‐target case. (p–t) Five‐target case. The distinct peaks in the range–velocity plane confirm that the frequency‐shifted echoes from different segments can be resolved as independently generated false targets, demonstrating the scalability of the proposed multi‐target illusion mechanism. All amplitudes are normalized to the experimentally measured reflection amplitude obtained under uniform full‐aperture modulation.

### 4.2 Scalability Characterization Through Segment‐Dependent Amplitude Control

We further investigate how the segment arrangement affects the reflection amplitude of the generated targets, as shown in Figure 5 and Supporting Information S1: Note 2 and Table S2. In Figure 5a–f, two frequency‐shifted channels with modulation frequencies of 0.5005 and 2.5005 MHz are used, and the velocity‐modulation component is kept identical so that the results correspond to slices at the same apparent velocity. The reflection amplitude of the single‐channel case in Figure 5a is defined as the normalization reference. When the metasurface aperture is divided into two segments, changing the column allocation directly redistributes the echo strength between the two corresponding targets. As listed in Supporting Information S1: Table S2, the normalized amplitude of T1 decreases from 0.77 to 0.38 when the number of columns allocated to its segment is reduced from 20 to 12, whereas the normalized amplitude of T2 increases from 0.09 to 0.39 when the number of columns allocated to its segment is increased from 4 to 12. This trend confirms that the target amplitude can be regulated by adjusting the segment size, because a larger segment contains more coherently modulated meta‐atoms and therefore provides a stronger frequency‐shifted echo component. Figure 5g–i further show the amplitude variation when the number of segments is increased. For the three‐, four‐, and five‐segment cases, the sums of the normalized peak amplitudes are approximately 0.72, 0.65, and 0.56, respectively, indicating that the peak‐amplitude deficit, defined here as 1 − Σ*α*_*q*_, increases as the aperture is divided into more independently modulated channels. Physically, when the aperture is subdivided into more segments, each segment contains fewer columns and its coherent aperture gain is reduced. Meanwhile, the finite phase coverage, voltage discretization, nonuniform amplitude response of the meta‐atoms, abrupt modulation discontinuities at segment boundaries, inter‐segment coupling, and finite FFT‐bin sampling can scatter part of the response into residual carrier components, higher‐order modulation harmonics, sidelobes, background fluctuations, or adjacent spectral bins. These nonideal factors become more pronounced for smaller segments, leading to a gradual decrease in the total peak amplitude as the number of segments increases.

**FIGURE 5:**
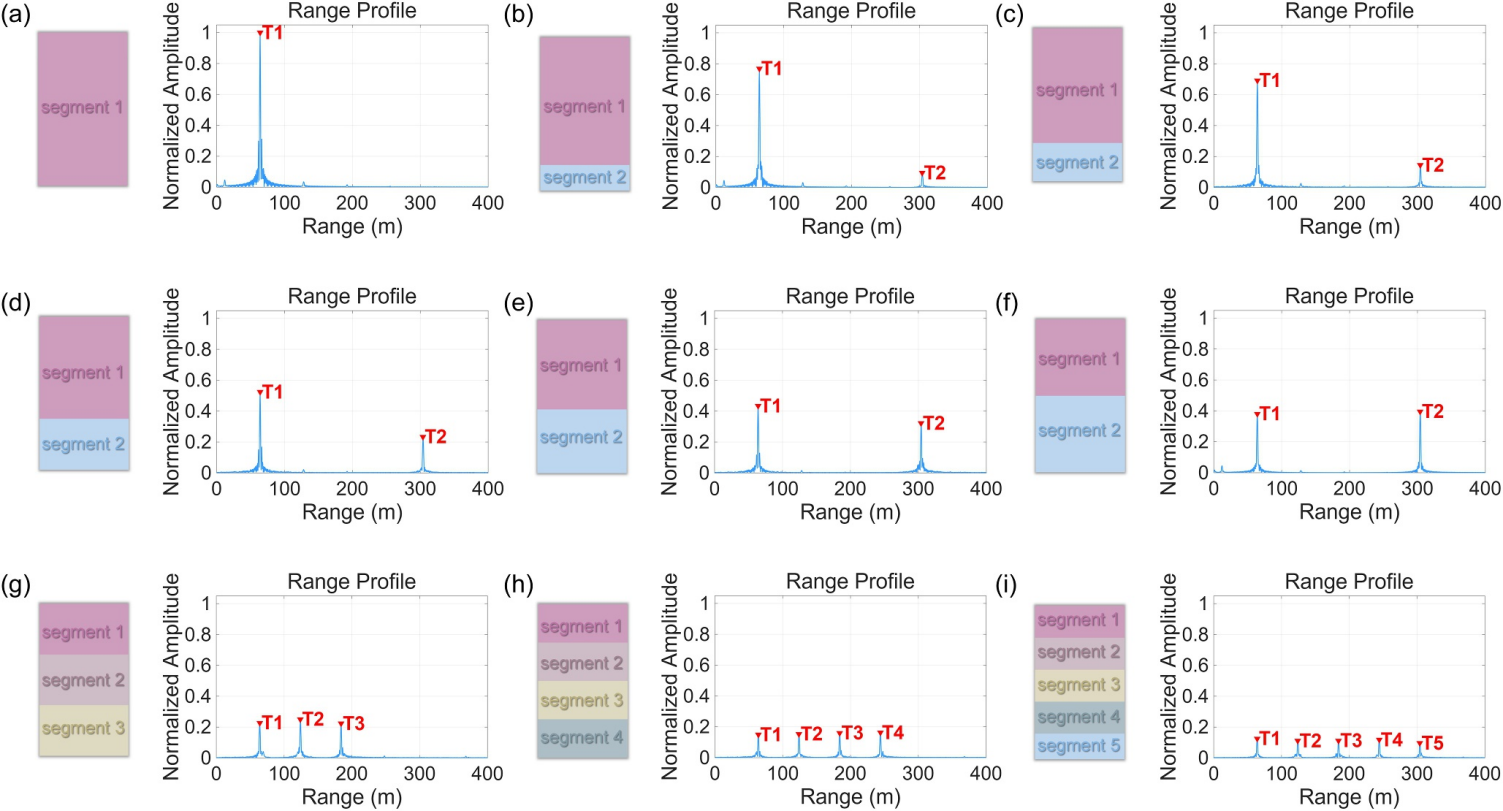
Influence of segment size and number of segments on the normalized reflection amplitude. Range profiles when changing the column allocation between two segments (a) 24:0, (b) 20:4, (c) 18:6, (d) 16:8, (e) 14:10, and (f) 12:12. The two frequency‐shifted channels are encoded by 0.5005 and 2.5005 MHz, respectively; only the 0.5005 MHz channel is used in (a). Range profiles when increasing the number of segments under the same velocity‐modulation setting. (g) 8:8:8, (h) 6:6:6:6, and (i) 5:5:5:5:4. All amplitudes are normalized to the reflection amplitude of T1 in (a).

To further evaluate the influence of these practical nonidealities, the corresponding simulation results under ideal conditions are provided in Supporting Information S1: Note 3. Despite the measured phase coverage of approximately 335°, rather than a complete 360°, the experimental results reproduce the target‐number scaling, designed range–velocity distributions, and segment‐dependent amplitude trends predicted by the simulations. The incomplete phase coverage mainly introduces residual carrier and higher‐order harmonic components, together with moderate amplitude deviations, but does not substantially affect the intended false‐target positions or the overall multi‐target illusion performance.

Taken together, the experiments in Figures 4 and 5 demonstrate two controllability dimensions of the proposed segmented spatiotemporal metasurface. Figure 4 and Supporting Information S1: Table S1 verify that independent temporal frequency coding can accurately determine the apparent range and velocity of multiple false targets, whereas Figure 5 and Supporting Information S1: Table S2 show that the segment size provides an additional handle for regulating their relative reflection amplitudes. Therefore, increasing the number of false targets requires not only additional independently addressable columns and control channels, but also sufficient effective aperture area for each segment to maintain an observable target response. In practice, the achievable number of false targets *N* is jointly limited by the column‐level addressability of the metasurface, the number of independent channels provided by the AWG and biasing system, and the actual conversion efficiency of each segment. The demonstrated cases should therefore be regarded as an experimental validation under the present aperture and control‐channel configuration, rather than the intrinsic upper limit of the proposed mechanism. When the aperture size, column‐level addressability, control‐channel count, and modulation fidelity are further improved, the number of resolvable false targets and their amplitude controllability are expected to increase correspondingly. Extending the present column‐addressable architecture to two‐dimensional spatial modulation would allow independent phase gradients to be encoded along both aperture axes, enabling beam steering in both azimuth and elevation. Combined with temporal spectral coding, this capability could extend the current range–velocity illusion to higher‐dimensional range–velocity–angle illusion generation.

## 5 Conclusion

In summary, this paper demonstrates a *scalable multi‐target illusion* strategy based on a *segmented spatiotemporal* metasurface and formulates its underlying operation as a *parallel‐sequence spectral translation mechanism*. By dividing one physical aperture into independently driven segments and loading different time‐varying bias sequences, the metasurface generates multiple parallel sequence‐coded spectral channels under the same LFM signal illumination. The fast‐time and slow‐time spectral components determine the apparent range and velocity of each false target, respectively, while segment allocation provides an additional degree of freedom for regulating the relative target amplitude. A tunable metasurface operating near 5 GHz was fabricated and tested using a USRP‐based LFM detection platform. Experimental two‐, three‐, four‐, and five‐target illusions were successfully demonstrated, and the measured features agreed well with theoretical predictions. Furthermore, amplitude‐controllable target generation was experimentally achieved by adjusting the segment size, providing customized energy allocation among different false targets. These results confirm that PSTM provides a unified mechanism for *scalable multi‐target illusion* generation, where the perceived target number, coordinates, and intensities are jointly governed by segment arrangement, temporal spectral coding, and aperture‐weighted scattering strength. Future work will focus on extending the present column‐wise segmentation to two‐dimensional aperture partitioning, providing additional spatial degrees of freedom for more flexible false‐target manipulation, while improving modulation fidelity, target‐intensity controllability, and real‐time adaptive control for dynamic scene reconstruction.

## Author Contributions

**Yujie Hong:** writing – original draft, conceptualization, methodology, software, data curation, formal analysis, supervision, validation, investigation, visualization, project administration, resources. **Dan Liu:** conceptualization, methodology, validation, investigation, visualization, formal analysis, writing – original draft. **Yi Qian:** writing – original draft, conceptualization, investigation, methodology, validation, visualization, formal analysis. **Guangzheng Ren:** visualization, investigation, validation. **Wenlong Li:** visualization, investigation, validation. **Hailin Huang:** visualization, investigation, validation. **Shangjie Chen:** visualization, investigation, validation. **Cheng Huang:** visualization, investigation, validation. **Huilin Mu:** writing – review and editing, conceptualization, methodology, project administration, formal analysis, data curation, supervision, resources. **Chunsheng Guan:** writing – review and editing, conceptualization, methodology, data curation, supervision, project administration, resources, funding acquisition. **Tong Cai:** writing – review and editing, conceptualization, methodology, software, data curation, formal analysis, supervision, validation, funding acquisition, project administration, resources, visualization.

## Conflicts of Interest

The authors declare no conflicts of interest.

## Supporting information


Supporting Information S1

## Data Availability

The data that support the findings of this study are available from the corresponding author upon reasonable request.
