# Supplementary material for: Scalable Multi‐Target Illusion via a Segmented Spatiotemporal Metasurface
Source: Nanophotonics. 2026 Oct 11;15(19):e70316. doi: 10.1002/nap2.70316 (PMC13655299; doi:10.1002/nap2.70316)
Supplement: Supplementary file 1 [file NAP2-15-e70316-s001.docx]

**Supplementary Information for**

**Scalable Multi-Target Illusion via a Segmented Spatiotemporal Metasurface**

Yujie Hong ^1, §^, Dan Liu ^1, §^, Yi Qian ^2, §^, Guangzheng Ren ^1^, Wenlong Li ^1^, Hailin Huang ^1^, Shangjie Chen ^1^, Cheng Huang ^1^, Huilin Mu ^1, *^, Chunsheng Guan ^1, *^, Tong Cai ^1, *^

*^1^ Air and Missile Defense College, Air Force Engineering University, Xi’an 710051, China.*

*^2^ Fundamentals Department, Air Force Engineering University, Xi’an 710051, China.*

*^§^ These authors contributed equally: Yujie Hong, Dan Liu, Yi Qian*

** Corresponding Authors: muhuilin@alu.hit.edu.cn (H. Mu), [chunshengguan@163.com](mailto:chunshengguan@163.com) (C. Guan),*

*caitong326@sina.cn (T. Cai)*

[Note 1. Theoretical Model	1](#_Toc238976266)

[Note 2. Experimental Data	5](#_Toc238976267)

[Note 3. Simulations and Comparison with Experimental Results	7](#_Toc238976268)

## Note 1. Theoretical Model

Consider an FMCW detector transmitting an LFM signal with carrier frequency *f_c_*, bandwidth *B*, chirp duration *T_p_*, and chirp slope

$$K=\frac{B}{T_{p}}$$

In the *n*-th chirp period, *nT_p_* ≤*t*< (*n*+1)*T_p_*, the transmitted signal can be written as

$$s_{\text{tx}}(t,n)=\exp\left\{j2\pi\left[f_{c}(t-nT_{p})+\frac{K}{2}(t-nT_{p})^{2}\right]\right\}$$

Here, *t* denotes the continuous time, *t*-*nT_p_* is the fast time within the *n*-th chirp, and *n* is the chirp index. For clarity, we first consider that the physical distance between the detector and the metasurface remains fixed within one chirp. If the physical distance is *R*_0_, the corresponding round-trip delay is

$$\tau_{0}=\frac{2R_{0}}{c}$$

where *c* is the speed of light in free space. For an unmodulated reflection, the received echo can be expressed as

$$s_{\text{rx}}(t,n)=\exp\left\{j2\pi\left[f_{c}(t-nT_{p}-\tau_{0})+\frac{K}{2}(t-nT_{p}-\tau_{0})^{2}\right]\right\}$$

After dechirp processing, the beat frequency is determined by *τ*_0_, and the measured range therefore corresponds to the physical distance *R*_0_.

To generate multiple target responses within one image, the metasurface aperture is segmented into *N* spatial segments, denoted by *Ω*_1_, *Ω*_2_, ..., *Ω*_N_. The *q*-th segment is driven by an independent time-varying bias sequence, which modulates the reflection phase of the tunable meta-atoms within that segment. The equivalent reflection coefficient of this segment can be expressed as

where *n* is the chirp index, and *ϕ_q_*(*t*, *n*) denotes the time-varying reflection phase. *α_q_* denotes the effective amplitude coefficient associated with the *q*-th segment. It is mainly determined by the aperture size of the segment, and also includes practical factors such as the reflection magnitude of the meta-atoms, modulation efficiency, and possible inter-segment coupling. In the ideal model, *α_q_* only weights the echo amplitude and does not affect the designed range or velocity. To control the range and velocity of the false target simultaneously, the phase modulation is designed to contain both fast-time and slow-time terms:

where *f_R_,_q_* is the range-modulation frequency, and fD,q is the velocity-modulation frequency. This equation indicates that each segment acts as an independent sequence-coded spectral-translation channel in the proposed parallel-sequence spectral translation mechanism (PSTM). The notation *f_m,q_* = *f_R_,_q_* + *f_D_,_q_* represents a composite modulation label containing the fast-time component *f_R_,_q_* and the slow-time component *f_D_,_q_*. Assigning different (*f_R_,_q_*, *f_D_,_q_*) pairs to different segments therefore translates the reflected spectrum into multiple segment-dependent frequency-shifted components on a single metasurface.

With the phase modulation introduced by the *q*-th segment, the modulated reflected signal becomes

$$s_{\text{ms},q}(t,n)=\Gamma_{q}(t,n)s_{\text{rx}}(t,n)$$

At the receiver, dechirp processing is performed by mixing the modulated echo with the conjugate of the transmitted signal, giving

$$s_{\text{IF},q}(t,n)=s_{\text{ms},q}(t,n)s_{\text{tx}}^{*}(t,n)$$

After expanding the expression and neglecting constant phase terms as well as higher-order weak coupling terms, the dominant phase of the IF component generated by the *q*-th segment can be written as

Equivalently, under the positive beat-frequency convention used for range extraction, the effective beat frequency is

$$f_{b,q}\approx K\tau_{0}+f_{R,q}$$

This relation corresponds to the spectral-translation stage of PSTM illustrated in Fig. 2(b). The fast-time modulation term *f_R_*_,_*_q_*(*t*-*nT_p_*) directly modifies the beat frequency after dechirp processing and is therefore manifested as a range displacement. Using the standard FMCW ranging relation *R*=*cf_b_*/(2*K*), the apparent range of the *q*-th false target is obtained as

$$R_{q}=R_{0}+\frac{cf_{R,q}}{2K}$$

This relation shows that a positive *f_R_*_,_*_q_* shifts the apparent target toward a larger range bin, whereas a negative *f_R_*_,_*_q_* shifts it toward a smaller range bin. Therefore, by properly selecting the fast-time modulation frequency *f_R_*_,_*_q_*, the false-target range can be flexibly tailored.

Velocity estimation, in contrast, relies on phase accumulation in the slow-time dimension. After a range FFT is performed for each chirp, the complex amplitude extracted at the *q*-th false-target range bin forms a slow-time sequence approximately given by

$$a_{q}[n]\approx\alpha_{q}\exp\left(j2\pi f_{D,q}t\right)$$

This sequence exhibits a linear phase progression from chirp to chirp, which is equivalent to a Doppler frequency. It should be emphasized that this Doppler component is not induced by actual mechanical motion of the metasurface, but by the pulse-to-pulse phase progression imposed by the time-varying reflection phase. According to the Doppler relation, the apparent radial velocity of the *q*-th false target is

$$v_{q}=\frac{\lambda f_{D,q}}{2}$$

where *λ*=*c*/*f_c_* is the operating wavelength. If the physical target or metasurface has a real radial velocity *v*_0_, the relation can be extended to *v_q_*=*v*_0_+*λf_D_*_,_*_q_*/2. Thus, *f_R_*_,_*_q_* and *f_D_*_,_*_q_* provides independent handles for positioning a false target along the range and velocity dimensions.

For the whole metasurface aperture, the spatiotemporal reflection response can be written as

$$\Gamma_{\text{PSTM}}(x,y,t,n)=\sum_{q=1}^{N} \chi_{q}(x,y)\alpha_{q}\exp\left\{j2\pi\left[f_{R,q}(t-nT_{p})+f_{D,q}t\right]\right\}$$

where *χ_q_*(*x*,*y*) is the spatial selection function of the *q*-th segment. It equals unity when (*x*,*y*)∈*Ω_q_* and zero otherwise. This relation gives the expression of PSTM: one physical scattering response is decomposed into multiple segment-resolved translated spectral components, and each component is assigned an independent spectral shift and amplitude weight. The detector receives the coherent superposition of all reflected components, whereas two-dimensional range-velocity processing separates them into different spectral locations.

The resulting range-velocity spectrum can therefore be approximated as a superposition of multiple target-like peaks:

$$S(R,v)\approx\sum_{q=1}^{N} \alpha_{q}P_{R}(R-R_{q})P_{V}(v-v_{q})$$

where *P_R_*(⋅) and *P_V_*(⋅) are the point-spread functions in the range and velocity dimensions, respectively. In the ideal case, *α_q_* scales with the effective aperture assigned to the corresponding segment, and denotes the effective intensity of the *q*-th false target. This intensity is mainly governed by the segment area. This relation indicates that PSTM allows a single physical metasurface to be reconstructed as multiple independent target responses in the range-velocity plane.

In practical design, the desired range-velocity features (*R_q_*, *v_q_*) are first specified, and the required modulation frequencies are obtained as

$$f_{R,q}=\frac{2K(R_{q}-R_{0})}{c}$$

$$f_{D,q}=\frac{2\left(v_{q}-v_{0}\right)}{\lambda}$$

These modulation frequencies are then used to generate the corresponding time-varying phase sequences, which are converted into executable bias sequences through the measured voltage-phase mapping of the tunable meta-atoms. By loading different voltage sequences onto different segments, multiple frequency-shifted echo channels are produced simultaneously under the same incident signal. After dechirp processing and two-dimensional fast Fourier transform shown in Fig. 2, these channels are resolved as multiple target-like peaks in the range-velocity plane. In this way, PSTM translates the response of a single programmable metasurface from one physical reflector into multiple segment-resolved spectral components, thereby enabling scalable multi-target illusion generation with controllable range, velocity, and amplitude features.

## Note 2. Experimental Data

**Table S1. Modulation parameters, theoretical values, and measured values for the multi-target illusion experiments in Fig. 4.**

| **Case** | **Fig.** | **Number of targets** | ***f_m,q_*** | | **theoretical range (m)** | **measured range (m)** | **theoretical velocity (m/s)** | **measured velocity (m/s)** |
| --- | --- | --- | --- | --- | --- | --- | --- | --- |
|  |  |  | ***f_R,q_* (MHz)** | ***f_D,q_* (kHz)** |  |  |  |  |
| Case 1 | Fig. 4  (a-e) | 2 | 0.5 | 1.0 | 64.12 | 63.0 | 30.00 | 30.09 |
|  |  |  | 1.0 | 1.0 | 124.12 | 123.0 | 30.00 | 30.09 |
| Case 2 | Fig. 4  (f-j) | 3 | 1.5 | 0.5 | 184.06 | 183.0 | 15.00 | 14.92 |
|  |  |  | 1.5 | 1.0 | 184.12 | 183.0 | 30.00 | 30.09 |
|  |  |  | 1.5 | 1.5 | 184.18 | 183.0 | 45.00 | 44.77 |
| Case 3 | Fig. 4  (k-o) | 4 | 0.5 | 0.8 | 64.10 | 63.0 | 24.00 | 23.22 |
|  |  |  | 1.0 | 1.6 | 124.19 | 123.0 | 48.00 | 48.19 |
|  |  |  | 1.5 | 2.4 | 184.29 | 184.2 | 72.00 | 72.16 |
|  |  |  | 2.0 | 3.2 | 244.38 | 244.2 | 96.00 | 96.14 |
| Case 4 | Fig. 4  (p-t) | 5 | 0.5 | 1.2 | 64.14 | 63.0 | 36.00 | 35.96 |
|  |  |  | 1.0 | 3.0 | 124.36 | 124.2 | 90.00 | 89.77 |
|  |  |  | 1.5 | 1.5 | 184.18 | 183.0 | 45.00 | 44.77 |
|  |  |  | 2.0 | 0.6 | 244.07 | 243.0 | 18.00 | 17.86 |
|  |  |  | 2.5 | 3.2 | 304.38 | 304.2 | 96.00 | 96.14 |

**Table S2. Segment settings and normalized reflection amplitudes for the segment-size and segment-number experiments in Fig. 5.**

| **Fig.** | **Number of segments** | **Target** | ***f_m,q_*** | | **Segment columns** | **Column ratio** | **Normalized amplitude** |
| --- | --- | --- | --- | --- | --- | --- | --- |
|  |  |  | ***f_R,q_* (MHz)** | ***f_D,q_* (kHz)** |  |  |  |
| Fig. 5(a) | 1 | T1 | 0.5 | 0.5 | 24 | 1.00 | 1.00 |
| Fig. 5(b) | 2 | T1 | 0.5 | 0.5 | 20 | 0.83 | 0.77 |
|  |  | T2 | 2.5 | 0.5 | 4 | 0.16 | 0.09 |
| Fig. 5(c) | 2 | T1 | 0.5 | 0.5 | 18 | 0.75 | 0.70 |
|  |  | T2 | 2.5 | 0.5 | 6 | 0.25 | 0.14 |
| Fig. 5(d) | 2 | T1 | 0.5 | 0.5 | 16 | 0.67 | 0.56 |
|  |  | T2 | 2.5 | 0.5 | 8 | 0.33 | 0.23 |
| Fig. 5(e) | 2 | T1 | 0.5 | 0.5 | 14 | 0.58 | 0.43 |
|  |  | T2 | 2.5 | 0.5 | 10 | 0.42 | 0.32 |
| Fig. 5(f) | 2 | T1 | 0.5 | 0.5 | 12 | 0.5 | 0.38 |
|  |  | T2 | 2.5 | 0.5 | 12 | 0.5 | 0.39 |
| Fig. 5(g) | 3 | T1 | 0.5 | 0.5 | 8 | 0.33 | 0.23 |
|  |  | T2 | 1.0 | 0.5 | 8 | 0.33 | 0.25 |
|  |  | T3 | 1.5 | 0.5 | 8 | 0.33 | 0.24 |
| Fig. 5(h) | 4 | T1 | 0.5 | 0.5 | 6 | 0.25 | 0.16 |
|  |  | T2 | 1.0 | 0.5 | 6 | 0.25 | 0.16 |
|  |  | T3 | 1.5 | 0.5 | 6 | 0.25 | 0.16 |
|  |  | T4 | 2.0 | 0.5 | 6 | 0.25 | 0.17 |
| Fig. 5(i) | 5 | T1 | 0.5 | 0.5 | 5 | 0.21 | 0.11 |
|  |  | T2 | 1.0 | 0.5 | 5 | 0.21 | 0.12 |
|  |  | T3 | 1.5 | 0.5 | 5 | 0.21 | 0.11 |
|  |  | T4 | 2.0 | 0.5 | 5 | 0.21 | 0.12 |
|  |  | T5 | 2.5 | 0.5 | 4 | 0.17 | 0.10 |

## Note 3. Simulations and Comparison with Experimental Results

Simulations were performed using the same LFM waveform by Matlab, physical distance, metasurface segmentation, modulation frequencies, sampling rate, coherent processing interval, and range-Doppler processing parameters as those used in the experiments. The simulated results corresponding to Figs. 4 and 5 are presented in Figs. S1 and S2, respectively, and the simulated normalized amplitudes in Fig. S2 are summarized in Table S3.

The experimental results reproduce the target-number scaling, range-velocity distributions, and segment-dependent amplitude trends predicted by the simulations. Compared with the simulated spectra in Fig. S1, the experimental spectra in Fig. 4 contains additional harmonic components and a higher background level. The harmonic components are mainly associated with the nonideal temporal reflection response of the fabricated metasurface. In particular, the measured phase coverage is approximately 335° rather than a complete 360°, and the reflection amplitude varies with the applied bias voltage. Consequently, the implemented reflection coefficient deviates from a constant-amplitude linear phase ramp, causing part of the reflected energy to be distributed into residual carriers and higher-order modulation components. The finite dynamic response of the varactor diodes and biasing circuits, together with waveform discretization, bias-voltage accuracy, and synchronization differences among the feeding channels, can introduce additional phase and amplitude errors at high modulation rates. The isolated clutter components observed in the measurements can also be attributed to environmental reflections, antenna coupling, cable and connector responses, system noise, and residual scattering from the experimental setup.

For the segment-size and segment-number cases, the experimental results in Fig. 5 follow the same amplitude-redistribution trend as the simulation results in Fig. S2. Reducing the number of columns assigned to a segment decreases the amplitude of its corresponding target, whereas increasing the allocated aperture increases the target amplitude. The experimental amplitudes do not vary strictly in proportion to the column ratios because the effective contribution of each segment is also affected by the voltage-dependent reflection magnitude, phase-calibration accuracy, finite modulation bandwidth, inter-segment coupling, and discontinuities at the segment boundaries. These nonideal effects become more noticeable when the aperture is divided into a larger number of smaller segments. Nevertheless, the measured target locations and the overall amplitude variation remain consistent with the simulated results, supporting the range-velocity and amplitude-control mechanisms of the segmented spatiotemporal metasurface.

**Figure S1.** **Simulated range-velocity responses corresponding to the experiments in Fig. 4.** (a, b) Two-target case; (c, d) three-target case; (e, f) four-target case; and (g, h) five-target case. Panels (a), (c), (e), and (g) show the three-dimensional range-Doppler maps, while panels (b), (d), (f), and (h) show the corresponding two-dimensional range-velocity response patterns. The signal, modulation, sampling, and processing parameters are identical to those used in the corresponding experiments.

**Figure S2. MATLAB-simulated influence of segment size and segment number on the normalized reflection amplitude, corresponding to the experiments in Fig. 5.** Simulated range profiles for column-allocation ratios of (a) 24:0, (b) 20:4, (c) 18:6, (d) 16:8, (e) 14:10, and (f) 12:12. The two frequency-shifted channels are encoded by range-modulation frequencies of 0.5005 and 2.5005 MHz, respectively, with the same velocity-modulation component; only the 0.5005 MHz channel is applied in (a). Simulated range profiles for (g) three segments with an 8:8:8 allocation, (h) four segments with a 6:6:6:6 allocation, and (i) five segments with a 5:5:5:5:4 allocation. All amplitudes are normalized to the amplitude of T1 in (a), and all simulation parameters are consistent with the corresponding experimental settings.

**Table S3. Segment settings and simulated normalized reflection amplitudes for the segment-size and segment-number cases in Fig. S2.**

| **Fig.** | **Number of segments** | **Target** | ***f_m,q_*** | | **Segment columns** | **Column ratio** | **Normalized amplitude** |
| --- | --- | --- | --- | --- | --- | --- | --- |
|  |  |  | ***f_R,q_* (MHz)** | ***f_D,q_* (kHz)** |  |  |  |
| Fig. S2(a) | 1 | T1 | 0.5 | 0.5 | 24 | 1.00 | 1.00 |
| Fig. S2(b) | 2 | T1 | 0.5 | 0.5 | 20 | 0.83 | 0.83 |
|  |  | T2 | 2.5 | 0.5 | 4 | 0.16 | 0.16 |
| Fig. S2(c) | 2 | T1 | 0.5 | 0.5 | 18 | 0.75 | 0.75 |
|  |  | T2 | 2.5 | 0.5 | 6 | 0.25 | 0.25 |
| Fig. S2(d) | 2 | T1 | 0.5 | 0.5 | 16 | 0.67 | 0.67 |
|  |  | T2 | 2.5 | 0.5 | 8 | 0.33 | 0.33 |
| Fig. S2(e) | 2 | T1 | 0.5 | 0.5 | 14 | 0.58 | 0.58 |
|  |  | T2 | 2.5 | 0.5 | 10 | 0.42 | 0.42 |
| Fig. S2(f) | 2 | T1 | 0.5 | 0.5 | 12 | 0.5 | 0.5 |
|  |  | T2 | 2.5 | 0.5 | 12 | 0.5 | 0.5 |
| Fig. S2(g) | 3 | T1 | 0.5 | 0.5 | 8 | 0.33 | 0.33 |
|  |  | T2 | 1.0 | 0.5 | 8 | 0.33 | 0.33 |
|  |  | T3 | 1.5 | 0.5 | 8 | 0.33 | 0.33 |
| Fig. S2(h) | 4 | T1 | 0.5 | 0.5 | 6 | 0.25 | 0.25 |
|  |  | T2 | 1.0 | 0.5 | 6 | 0.25 | 0.25 |
|  |  | T3 | 1.5 | 0.5 | 6 | 0.25 | 0.25 |
|  |  | T4 | 2.0 | 0.5 | 6 | 0.25 | 0.25 |
| Fig. S2(i) | 5 | T1 | 0.5 | 0.5 | 5 | 0.21 | 0.21 |
|  |  | T2 | 1.0 | 0.5 | 5 | 0.21 | 0.21 |
|  |  | T3 | 1.5 | 0.5 | 5 | 0.21 | 0.21 |
|  |  | T4 | 2.0 | 0.5 | 5 | 0.21 | 0.21 |
|  |  | T5 | 2.5 | 0.5 | 4 | 0.17 | 0.17 |
